# CT derived left atrial size identifies left heart disease in suspected pulmonary hypertension: Derivation and validation of predictive thresholds

**DOI:** 10.1016/j.ijcard.2018.02.114

**Published:** 2018-06-01

**Authors:** Benjamin J. Currie, Chris Johns, Matthew Chin, Thanos Charalampopolous, Charlie A. Elliot, Pankaj Garg, Smitha Rajaram, Catherine Hill, Jim W. Wild, Robin A. Condliffe, David G. Kiely, Andy J. Swift

**Affiliations:** a Infection, Immunity and Cardiovascular Disease, University of Sheffield, Sheffield, UK; b Sheffield Pulmonary Vascular Disease Unit, Royal Hallamshire Hospital, Sheffield, UK; c Radiology Department, Royal Hallamshire Hospital, Sheffield, UK; d INSIGNEO, Institute for in silico medicine, University of Sheffield, UK

**Keywords:** Pulmonary hypertension, Left heart disease, Left atrial area, Left atrial volume, Computed tomography pulmonary angiography, Magnetic resonance imaging

## Abstract

**Background:**

Patients with pulmonary hypertension due to left heart disease (PH-LHD) have overlapping clinical features with pulmonary arterial hypertension making diagnosis reliant on right heart catheterization (RHC). This study aimed to investigate computed tomography pulmonary angiography (CTPA) derived cardiopulmonary structural metrics, in comparison to magnetic resonance imaging (MRI) for the diagnosis of left heart disease in patients with suspected pulmonary hypertension.

**Methods:**

Patients with suspected pulmonary hypertension who underwent CTPA, MRI and RHC were identified. Measurements of the cardiac chambers and vessels were recorded from CTPA and MRI. The diagnostic thresholds of individual measurements to detect elevated pulmonary arterial wedge pressure (PAWP) were identified in a derivation cohort (n = 235). Individual CT and MRI derived metrics were tested in validation cohort (n = 211).

**Results:**

446 patients, of which 88 had left heart disease. Left atrial area was a strong predictor of elevated PAWP>15 mm Hg and PAWP>18 mm Hg, area under curve (AUC) 0.854, and AUC 0.873 respectively. Similar accuracy was also identified for MRI derived LA volume, AUC 0.852 and AUC 0.878 for PAWP > 15 and 18 mm Hg, respectively. Left atrial area of 26.8 cm^2^ and 30.0 cm^2^ were optimal specific thresholds for identification of PAWP > 15 and 18 mm Hg, had sensitivity of 60%/53% and specificity 89%/94%, respectively in a validation cohort.

**Conclusions:**

CTPA and MRI derived left atrial size identifies left heart disease in suspected pulmonary hypertension with high specificity. The proposed diagnostic thresholds for elevated left atrial area on routine CTPA may be a useful to indicate the diagnosis of left heart disease in suspected pulmonary hypertension.

## Highlights

• Routine CTPA can diagnose left heart disease in suspected pulmonary hypertension.
• Complex multiparameter models do not improve LHD diagnosis.
• Highly specific thresholds have been derived and validated.

## 1 Background

Pulmonary hypertension is a complex condition with multiple underlying causes. It is defined on right heart catheterisation (RHC) as a resting mean pulmonary arterial pressure (mPAP) greater than or equal to 25 mm Hg, Of the 5 major World Health Organization (WHO) classifications of Pulmonary Hypertension Group 2 pulmonary hypertension due left heart disease (LHD) is the most common [1]. Unlike pulmonary arterial hypertension (PAH) there are limited treatment options in pulmonary hypertension due to left heart disease (PH-LHD) [2,3]. A number of PAH approved therapies have proven to have no benefit or to be detrimental to a patient's survival when trialled in PH-LHD [[4], [5], [6]], thus treatment is focused on treating heart failure [3,5]. Despite this, there is significant interest in treatment of selected groups of patients in clinical trials [[7], [8], [9], [10]].

A pulmonary arterial wedge pressure (PAWP) > 15 mm Hg alongside mPAP > 25 mm Hg is used to define PH-LHD. Both of these are measured on RHC, an invasive test with a 1% risk of serious complications. In some instances PAWP has been shown to be poor indicator for Group 2 PH-LHD [11]. Some patients with PAWP 16–18 mm Hg have been shown to have clinical features more suited to PAH [12]. Reliance on RHC for diagnosis, often the final investigation in the diagnostic algorithm, results in increased risk to patients, a burden on resources and loss of patients' time through referrals to tertiary centres [1].

CT pulmonary angiography is used routinely in the diagnostic algorithm for pulmonary hypertension [1]. CT measurements such as increased main pulmonary artery diameter and pulmonary artery to aortic ratio have shown to predict the presence of pulmonary hypertension [13] and PAH [14]. CT measurements including left atrial size have proven useful in identifying patients with PH-LHD [[15], [16], [17]]. However, no study has both derived and tested predictive thresholds of CT metrics in separate derivation and validation cohorts to identify elevated PAWP.

Therefore, the aim of the present study was to assess how CT pulmonary angiography derived metrics in isolation and as part of a derived model, could be used to predict the presence of elevated PAWP. A further aim was to assess the utility of CT measurements in comparison to magnetic resonance imaging (MRI) imaging derived variables.

## 2 Methods

### 2.1 Patients

Consecutive patients with suspected pulmonary hypertension who underwent baseline MRI and CT between 24th April 2012 and 30th March-2016 were identified from the ASPIRE registry [18] (Assessing the Spectrum of Pulmonary hypertension Identified at a Referral Centre). Patients were required to have MRI and CT within 3 months, and a diagnostic RHC. Sub-analysis was performed in the patients with suspected pulmonary arterial hypertension or left heart disease. For this, patients with CT pulmonary angiogram evidence of acute or chronic pulmonary emboli, at least moderate parenchymal lung disease or known diagnosis with group 5 pulmonary hypertension were excluded. Ethical approval was granted from a local ethics committee for this retrospective study and written consent was waived (ref c06/Q2308/8).

### 2.2 CT acquisition

CT pulmonary angiograms were performed either at the regional centre prior to transfer or at the Sheffield Pulmonary Vascular Disease Unit. CT pulmonary angiograms took place in 66 different hospitals from Wales, Midlands and the north of England. The majority of scans took place in Sheffield (76.5%), the tertiary referral centre.

Sheffield CT pulmonary angiograms were performed on a 64-slice MDCT scanner (Light-Speed General Electric Medical Systems, Milwaukee, WI). The images studies were not cardiac gated. Standard acquisition parameters were used: 100 mA utilising automated dose reduction, 120 kV, pitch 1, rotation time of 0.5 s and 0.625 mm collimation. The field of view was 400 × 400 mm and the acquisition matrix was 512 × 512. 100 ml of intravenous contrast agent (Ultravist 300; Bayer Schering, Berlin, Germany) was administered at 5 ml/s. HRCT images were also reconstructed from the contrast-enhanced acquisitions using 1.25 mm collimation from the apex of the lung to the diaphragm. For all studies slice thickness and number of slices were recorded. Limits were set at minimum slice number of 50 and maximum slice thickness of 5 mm. Across the studies slice thickness ranged from 0.5 mm to 5 mm with the mean slice thickness being 0.765 mm (SD 0.485). The minimum/maimium number of slices was 55/1001 with mean 434 (SD 117). Patients were excluded from the study if the images were not of diagnostic quality as graded by a chest radiologist.

### 2.3 MR acquisition

CMR imaging was performed on a 1.5 T whole body scanner GE HDx (GE Healthcare, Milwaukee, USA), using an 8-channel cardiac-coil, in supine position. Long axis LV 2-chamber and 4-chamber CINE views were acquired. 4 chamber and short axis (SA) cine images were acquired using a retrospective cardiac gated multi-slice balanced steady state free precession (bSSFP) sequence. A stack of axial images in the SA plane with slice thickness of 8–10 mm were acquired, fully covering both ventricles from base to apex. The bSSFP sequence parameters were: TR 2.8 ms, TE 1.0 ms, Flip angle of 50°, FOV = 48 × 43.2, 256 × 256 matrix, 125 kHz bandwidth and slice thickness of 10 mm.

### 2.4 CT image analysis

**Fig. 1:**
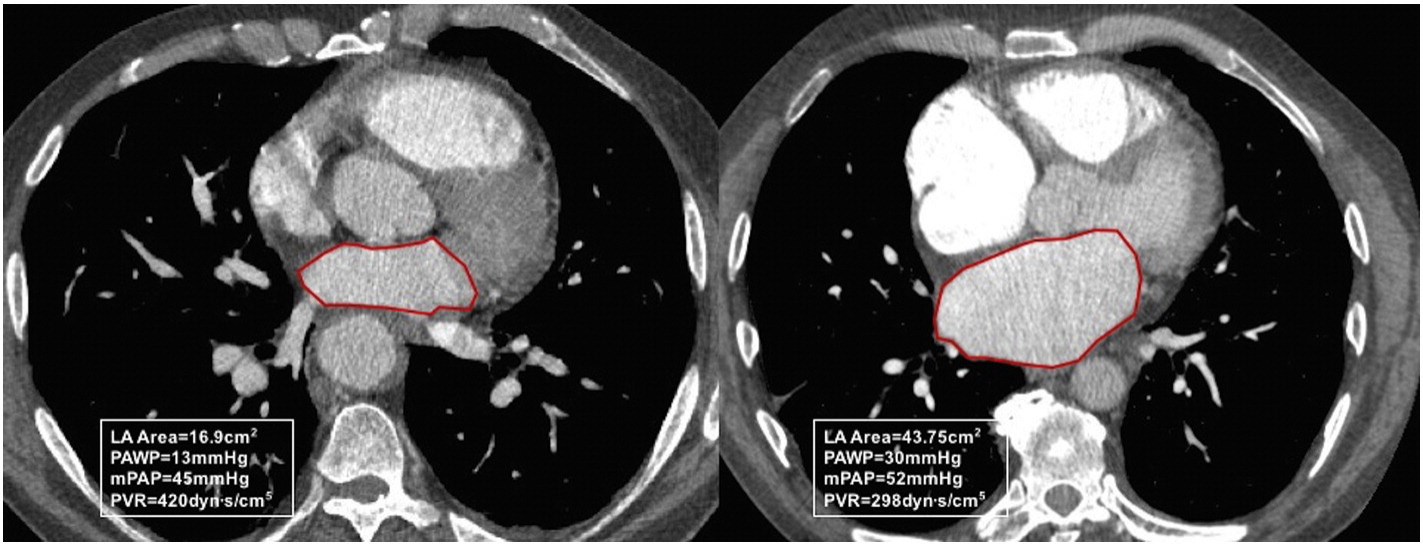
LA area region of interests in a patient with idiopathic pulmonary arterial hypertension (left) and a patient with pulmonary hypertension associated with left heart disease (right).

Image analysis was carried out with the observer blinded to right heart catheter data. A detailed analysis was performed including; area measurement of the 4 cardiac chambers were made on axial images. The measurements were taken from the image where the chamber visually appeared largest. On the image showing the maximal left atrial area, the left atrial area was manually traced and the pulmonary veins and the atrial appendage were excluded. For left atrial area the anterior-posterior diameter was also recorded. Fig. 1 shows an example of the measurement of the left atrium. For ventricular measurements the atrio-ventricular (AV) valves (mitral and tricuspid respectively) were visible on the slice. The valve and mid transverse dimension were recorded for both ventricles on the same slice as the area, along with the respective ventricular muscle areas. Maximal right atrial area and transverse and longitudinal diameters were recorded.

Cardiac vessel measurements; main pulmonary artery (MPA) diameter, superior vena cava (SVC) area, and ascending and descending aorta diameters were all recorded. These measurements were recorded on the slice where the MPA appeared widest and both main branches of the pulmonary artery were visible. Right and left main pulmonary artery diameters were measured on separate slices were each appeared widest and roughly 1 cm from the bifurcation. Pulmonary veins were measured separately at the respective widest points as they enter the left atrium. A reconstructed 4 chamber view was created from each set of scans and the maximal area of each cardiac chamber was measured. All diametric vessel measurements were made perpendicular to the direction of the vessel. All length measurements were recorded in centimetres (cm) and all area measurements in centimetres squared (cm^2^).

### 2.5 MR image analysis

Image analysis was undertaken on a GE Advantage Workstation 4.4 and GE Advantage Workstation ReportCard. The MR observer was blinded to the patient clinical information and cardiac catheter parameters. Scans were defined as non-diagnostic when image quality significantly affected cardiac measurements or, volumetric analysis could not be accurately performed. LA volume was estimated using the well-established bi-plane area-length method [19,20]. LV long axis (two chamber view) and four chamber views were analysed. MRI LA measurements were taken at end-ventricular systole, equating to maximal atrial size.LA volume was calculated from the relation: (0.85 × LA area two chamber view × LA area four chamber view) / ((LA length two chamber + LA length four chamber) / 2). The CMR parameters were corrected where appropriate for body surface area (BSA), as previously reported in the literature [21,22].

### 2.6 Statistics

Histograms were constructed for the CT metrics to assess for normal distribution. Mean and standard deviation were used for normally distributed data. Where appropriate Pearson correlation coefficients were calculated for CT-derived metrics versus PAWP. Categorical and continuous data were compared between the PAWP ≤ 15 mm Hg and PAWP > 15 mm Hg groups using Chi-square and independent *t*-test respectively. The patient cohort was split into two randomly constructed cohorts (derivation and validation). These two cohorts were also compared using *t*-tests and Pearson Chi-square tests.

In the derivation cohort, candidate differentiators predictive at univariate analysis with *p* < 0.05 were entered into a binary logistic regression to detect PAWP > 15 mm Hg using forward selection. Receiver operating characteristic (ROC) analysis was performed to determine diagnostic accuracy of candidate CT metrics with area under the ROC curve (AUC) results presented. Chi-square and Fishers exact test were used to calculate sensitivity, specificity, positive and negative predictive values. Predictive thresholds for univariate CT metrics with high specificity were chosen for identification of elevated PAWP (both PAWP > 15 and PAWP > 18) in the derivation cohort and were tested in the validation cohort. MRI derived left atrial volume was correlated with CT metrics and discriminatory ability of CT and MRI at ROC analysis compared. For these same variables, ROC analysis was undertaken in the whole cohort to predict how well these MRI-derived variables discriminated increased PAWP > 15 mm Hg and >18 mm Hg. 15 patients were analysed separately by two observers to determine interobserver variability and one of the observers repeated analysis on 15 patients to assess for intraobserver variability.

All statistics were performed in SPSS 21 (IMB, Chicago) and all graphs were produced using GraphPad, Prism (GraphPad, San Diego). A *p*-value of < 0.05 was considered statistically significant.

## 3 Results

Four-hundred forty-six (446) patients with suspected pulmonary hypertension were identified who underwent MRI, CT and RHC (including PAWP measurement) within period studied. 381 patients had pulmonary hypertension. Mean age 64.0 (SD 13), 170 Males: 276 Females. Table 1, shows demographic data and CT derived measurements for the study and compares those with PAWP > 15 mm Hg to those with PAWP ≤ 15 mm Hg. The mean interval between CT and RHC was 18 days (SD 44). 231 patients were identified for subgroup analysis with suspected pulmonary arterial hypertension or pulmonary hypertension owing to left heart disease.

**Table 1:** Demographics and CTPA derived metrics.

		PAWP ≤ 15 mm Hg n = 358	PAWP > 15 mm Hg n = 88	P-value
Age (years)		62.5 (13.4)	70 (8.7)	<0.001
Sex	F	222 (62%)	54 (61.4%)	0.911
	M	136 (38%)	34 (38.6%)	
Body surface area (m^2^)		1.82 (0.24)	1.87 (0.21)	0.099
WHO functional class	I	0	1 (1.1%)	0.073
	II	49 (14%)	8 (9.1%)	
	III	267 (76.5%)	74 (84.1%)	
	IV	33 (9.5%)	5 (5.7%)	
ISWT walking distance (m)		231 (201)	183 (160)	0.057
Right heart catheter	mPAP (mm Hg)	40 (15)	41 (12)	0.609
	mRAP (mm Hg)	9 (5)	14 (5)	<0.001
	PAWP (mmHg)	11 (3)	21 (5)	<0.001
	SVO_2_ (%)	65.4 (8.4)	64.7 (9.3)	0.514
	PVR (dyn·s/cm^5^)	572 (409)	354 (268)	<0.001
	CO (L/min)	4.91 (1.59)	4.99 (1.46)	0.662
	CI (L/min/m^2^)	2.70 (0.84)	2.68 (0.76)	0.782
RV area (cm^2^)		28.3 (9.39)	28.7 (8.92)	0.747
RV muscle area (cm^2^)		3.92 (1.76)	3.75 (1.72)	0.404
RA area (cm^2^)		28 (10.9)	35.5 (1.30)	<0.001
LV area (cm^2^)		20.9 (6.86)	24.4 (8.03)	<0.001
LV muscle area (cm^2^)		15.5 (4.06)	17.3 (5.54)	<0.001
LA area (cm^2^)		19.6 (5.88)	29.5 (8.06)	<0.001
LA anterior-posterior diameter (cm)		3.68 (0.78)	4.79 (0.83)	<0.001
MPA diameter (cm)		3.15 (0.58)	3.21 (0.50)	0.397
Ascending aorta diameter (cm)		3.1 (0.43)	3.32 (0.47)	<0.001
SVC area (cm^2^)		3.54 (1.22)	4.41 (1.52)	<0.001
Total PV area (cm^2^)		4.72 (1.85)	6.19 (2.48)	<0.001
Total PV/MPA area ratio		0.65 (0.32)	0.79 (0.32)	<0.001
RV/LV area ratio		1.52 (0.80_	1.29 (0.58)	0.011
RV/LV muscle area ratio		0.27 (0.14)	0.23 (0.12)	0.028
RA/LA area ratio		1.51 (0.67)	1.22 (0.38)	<0.001
RV/LA area ratio		1.55 (0.67)	1.02 (0.39)	<0.001
Coronary calcification	Y	157 (43.9%)	57 (64.8%)	0.002
	N	200 (55.9%)	31 (35.2%)	
Aortic annulus calcification	Y	80 (22.3%)	33 (37.5%)	0.013
	N	277 (77.4%)	55 (62.5%)	
Mitral annulus calcification	Y	35 (9.8%)	22 (25%)	<0.001
	N	322 (89.9%)	66 (75%)	

Abbreviations: WHO-World Health Organisation, mPAP-mean Pulmonary Artery Pressure, mRAP-mean Right Atrial Pressure, PAWP-Pulmonary Arterial Wedge Pressure, SVO_2_-Mixed Venous Oxygen Saturation, PVR-Pulmonary Vascular Resistance, CO-Cardiac Output, CI-Cardiac Index, ISWT-Incremental Shuttle Walking Test, RV-Right Ventricle, RA-Right Area, LV-Left Ventricle, LA-Left Atrial, MPA-Main Pulmonary Artery, SVC-Superior Vena Cava, PV-Pulmonary Vein.

### 3.1 Association to invasive PAWP

**Fig. 2:**
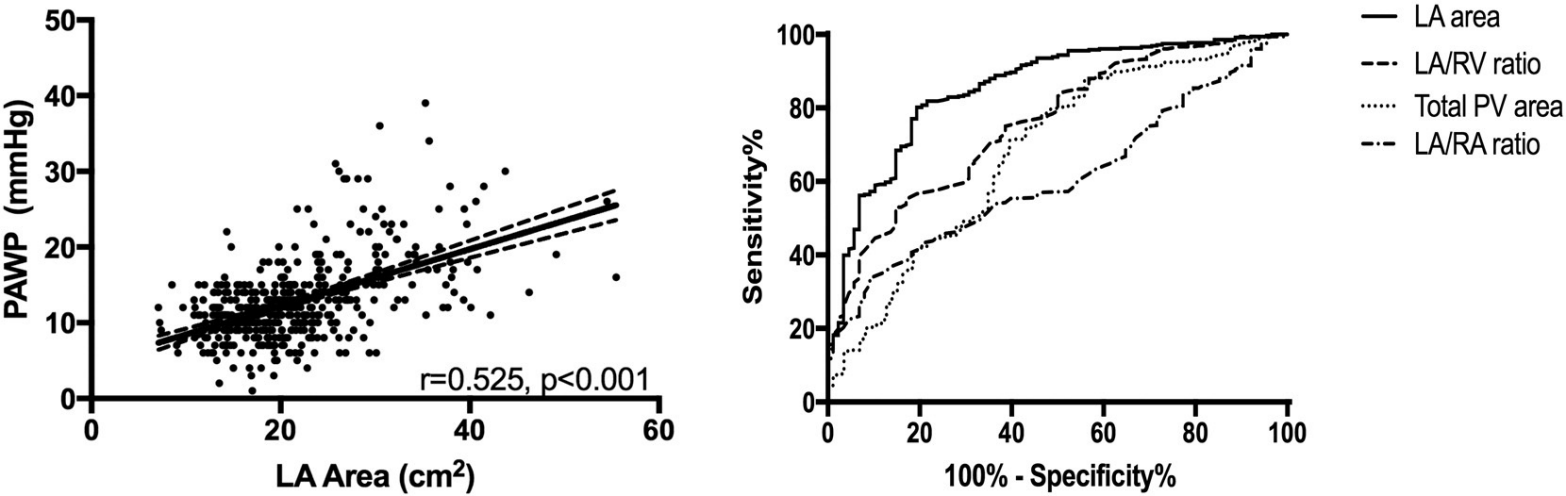
Correlation of left atrial (LA) area and pulmonary arterial wedge pressure (PAWP), left. And right, receiver operating characteristic curves for LA area, LA/RV area, Total PV area and LA/RA area ratio, AUC 0.854, 0.757, 0.681 and 0.601, respectively.

Left atrial area showed the strongest correlation with PAWP, *r* = 0.525, *p* < 0.001 a scatter plot is provided Fig. 2. The following metrics also correlated with PAWP; left atrial anterior posterior diameter, *r* = 0.509, *p* < 0.001, total pulmonary vein area, *r* = 0.323, *p* < 0.001, RV/LA area ratio, *r* = −0.322, *p* < 0.001, left ventricular muscle area, *r* = 0.230, *p* < 0.001, right atrial area, *r* = 0.225, *p* < 0.001 and left ventricular area, *r* = 0.221, *p* < 0.001. MRI derived LA volume correlated most strongly with CT derived LA area, *r* = 0.836, *p* < 0.001. 4 chamber LA area reconstructed had a marginally weaker correlation with LA volume on MRI, *r* = 0.815, *p* < 0.001. All measured variables were normally distributed.

### 3.2 Group comparisons and regression

Supplementary Table 1 presents the demographic data for the derivation and validation cohorts. There was no significant difference in the demographics between the cohorts. Fig. 2 presents ROC curves of 4 key CT derived variables and in the whole patient cohort; LA area AUC = 0.854, *p* < 0.001, LA/RV area ratio AUC = 0.757, *p* < 0.001, Total PV area AUC = 0.681, *p* < 0.001, LA/RA area ratio AUC = 0.601, *p* = 0.002.

The results of multivariate analysis for differentiating PAWP≤15 mmHg and PAWP > 15 mm Hg showed left atrial area was the only independent predictor of elevated PAWP (*p* < 0.001), with no additive value of other CT metrics.

In Table 2 the results of ROC analysis in validation cohort of CT and MRI derived left atrial measurements using both 15 mm Hg and 18 mm Hg as limits for elevated PAWP.

**Table 2:** Results of ROC analysis in validation cohort for LA measurements in CT and MRI derived variables at PAWP>15 mm Hg and PAWP>18 mm Hg.

	Area under curve (Standard deviation)	P-value
*CT metrics for PAWP > 15 mm Hg*		
LA area	0.858 (0.034)	<0.001
LA area BSA indexed	0.848 (0.037)	<0.001
LA anterior-posterior diameter	0.834 (0.035)	<0.001

*MRI metrics for PAWP > 15 mm Hg*		
LA 2ch area	0.833 (0.037)	<0.001
LA 4ch area	0.851 (0.035)	<0.001
LA volume	0.852 (0.036)	<0.001
LA volume BSA indexed	0.843 (0.041)	<0.001

*CT metrics for PAWP > 18 mm Hg*		
LA area	0.873 (0.036)	<0.001
LA area BSA indexed	0.855 (0.042)	<0.001
LA anterior-posterior diameter	0.842 (0.038)	<0.001

*MRI metrics for PAWP > 18 mm Hg*		
LA 2ch area	0.868 (0.034)	<0.001
LA 4ch area	0.857 (0.038)	<0.001
LA volume	0.878 (0.034)	<0.001
LA volume BSA indexed	0.865 (0.041)	<0.001

Abbreviations: PAWP-Pulmonary Arterial Wedge Pressure, LA-Left Atrium, BSA-Body Surface Area, CT-Computed Tomography, MRI-Magnetic Resonance Imaging.

Specific thresholds for left atrial area identified in the derivation cohort through assessment of the ROC coordinate plots. The thresholds identified were 26.8 cm^2^ and 30 cm^2^ for PAWP > 15 mm Hg and 18 mm Hg respectively. Supplementary Table 3 shows the sensitivities and specificities of all 4 thresholds when tested in the respective validation cohorts. In the validation cohort 26.8 cm^2^ were 89% specific, sensitivity was marginally higher at 60% for detecting PAWP > 15, versus 53% for detecting PAWP > 18.

MRI derived left atrial volume was the most accurate MRI variable for predicting PAWP > 15 (AUC = 0.852, *p* < 0.001), similar to CT-derived LA area AUC = 0.858, *p* < 0.001. 4-Chamber reconstructed LA area had an AUC of 0.805, *p* < 0.001 in the validation cohort which was inferior to LA area measured axially.

### 3.3 Subgroup analysis

In the 231 patients with suspected PAH or left heart disease, the accuracy for predicting PAWP > 18 for left atrial area was higher with area under the curve 0.907 (*p* < 0.001) versus PAWP > 15 for which the area under the curve was 0.874 (*p* < 0.001). Baseline demographics for the subgroup derivation and validation cohorts are shown in Supplementary Table 2 which demonstrates no significant difference between the two cohorts. The thresholds of 27.1 cm^2^ and 30.3 cm^2^ for 15 and 18 respectively found from the derivation cohort in the subgroup analysis. The subgroup analysis showed higher specificity particularly in the PAWP > 18 mm Hg analysis with specificity 95%. Sensitivity was marginally higher at 65% for detecting PAWP > 15, versus 60% for detecting PAWP > 18.

### 3.4 Reproducibility

Inter-observer reproducibility for the key variables is shown in Supplementary Table 4. Left atrial area and left atrial AP dimension showed excellent inter-observer reproducibility, ICC = 0.972, *p* < 0.001 and ICC = 0.921, *p* < 0.001 respectively. Intra class correlation for left atrial was 0.963, *p* < 0.001.

## 4 Discussion

CT derived left atrial area is a strong and independent predictor of increased PAWP in patients with suspected pulmonary hypertension. Enlarged left atrial area using thresholds of 26.8 cm^2^ and 30.0 cm^2^ to predict Group 2 pulmonary hypertension due to PAWP > 15 mm Hg and PAWP > 18 mm Hg respectively had a specificity of 89% and 94% in a separate validation cohort. In a subgroup analysis of only those patients with suspected PAH and PH-LHD, to identify PAWP>18 mm Hg a 30.3 cm^2^ threshold for LA area was 95% specific. The study demonstrates increased PAWP can be identified to a high degree of specificity using CT derived LA area and may support the diagnosis of left heart disease in suspected pulmonary hypertension alongside clinical assessment. When left atrial area was measured using 4-chamber reconstruction the area under the curve was marginally inferior to when measured axially, one possible explanation is error in identifying an optimal 4 chamber view on reconstruction.

In this study of 446 patients with suspected pulmonary hypertension, we show that left atrial (LA) area is the most predictive CT measurement for the detection of a raised pulmonary artery wedge pressure. A threshold of 30 cm2 had 94% specificity to detect elevated PAWP >18 mm Hg in the full validation cohort. Previously, identified candidate LA markers: LA/right atrium ratio, LA/right ventricle and LA/body surface area, were all of weaker diagnostic accuracy compared to LA area. A regression model comprising a number of CT metrics did not add diagnostic value over LA area alone. When patients with chronic thromboembolic disease, at least moderate lung disease and known miscellaneous causes of PH were excluded the accuracy marginally for detection of PAWP > 15 and PAWP > 18 respectively. CT derived LA area was equally accurate for predicting elevated PAWP compared to MRI derived left atrial volume. The inter-observer correlation of LA area was excellent.

LA area has previously been shown to predict the presence of elevated PAWP in selected populations [[15], [16], [17]]. Safdar et al. [15], showed a correlation of *r* = 0.45 was between LA area and PAWP, marginally weaker than in the present study. Katikreddy et al. [16], found LA area to be a predictor of the presence of LHD in PH, with area under the curve at ROC analysis of 0.76 compared with 0.85 in the present study. Juivraj et al. [17] found the area under curve for LA area to predict PH in LHD was also lower at 0.73.

Thresholds for enlarged LA area to identify elevated PAWP have previously been identified, these range from 20 cm^2^ to 31 cm^2^ [[15], [16], [17]]. Until now, no studies have validated these derived thresholds in separate cohorts. In this study, highly specific thresholds were sought, and LA area of 26.8 cm^2^ and 30.0 cm^2^ were identified to predict PAWP > 15 mm Hg and PAWP>18 mm Hg, respectively. For predicting elevated PAWP > 15 in the validation cohort there was a sensitivity of 60% and specificity of 89%. In the 30.0 cm^2^ threshold for PAWP > 18 mm Hg sensitivity was 53% and specificity 94% in the validation cohort. Safdar et al. [15] chose 30 cm^2^ as a threshold, for which sensitivities and specificities were not calculated but this was highly accurate on ROC analysis, with an area under the curve of 0.996. Jivraj et al. [17], selected a left atrial area of 24 cm^2^, prioritising specificity over sensitivity, with a the sensitivity was 44% and specificity 93%. A threshold of 20 cm^2^ was selected by Katikreddy et al. [16]. Sensitivity and specificity were not recorded at this threshold as instead they were calculated by combining LA area > 20 cm^2^ with normal RV area and were 77% and 94%, respectively. However, in the present study the LA area/RV area ratio was of weaker accuracy than LA area alone, AUC = 0.757 versus AUC = 0.854 respectively. We postulate that RV size does not aid the differentiation of pre and post capillary pulmonary hypertension given that the RV can increase in size in both conditions to a variable degree. Prior studies have not directly compared sensitivity/specificity analysis of CT measured LA area alone versus LA area adjusted for RV. LA area/RA area has also been shown to have diagnostic value and it is postulated correcting for RA area will account for the variation in volume of the LA during the cardiac cycle (INSERT reference here 27573596 international journal of cardiology, as per reviewer 1). However in the present study LA area alone despite the potential inaccuracy due to variation with cardiac phase was the most predictive measurement for the diagnosis of left heart disease.

CTPA has high sensitivity and specificity in detecting thromboembolic disease and shown to be of equal benefit in diagnosis as V/Q scintigraphy [23]. Moderate lung disease can be assessed and identified using pulmonary function tests, X-ray and CT findings [24]. A number of haematological, systemic and metabolic conditions are associated with miscellaneous Group 5 PH^25^. In the subgroup analysis, excluding patients with known clinical and CT features of chronic thromboembolic disease, at least moderate lung disease or a known diagnosis that meets criteria for group 5 PH, we found marginally improved the effectiveness of LA area to identify patients for increased PAWP. This represents a clinically relevant diagnostic dilemma, where it may be challenging to differentiate PAH from PH-LHD.

Using a threshold of 30.3 cm to identify PAWP > 18 mm Hg gave a specificity of 95% in the validation cohort. This may represent a clinically relevant complementary tool to aid the referral pathway and reduce unnecessary referrals of patients with significantly elevated left ventricular filling pressure.

An advantage of this study is the use of derivation and validation cohorts to produce predictive thresholds using ROC analysis and then determine their accuracy in a separate, independent cohort. In all previous studies the defined limit for elevated PAWP was >15 mm Hg as defined in the recent guidelines [24]. In this study, the thresholds of enlarged LA area were set using a higher limit of PAWP > 18 mm Hg, as well as PAWP > 15 mm Hg. Studies have shown that there are a number of patients with PAH that have elevated PAWP between 15 and 18 mm Hg [12,26]. In the ASPIRE registry study, 20 patients were shown to have a clear phenotype of pulmonary arterial hypertension (PAH) but their PAWP was between 16 and 18 mm Hg^18^.

Specificity was prioritised over sensitivity, as it was the aim to identify those patients with left heart disease before right heart catheter to prevent unnecessary investigations and management. The borderline patients and those who more likely had other forms of pulmonary hypertension would not be identified due to lower sensitivity and would require further investigations as we would not wish to exclude these patients from potential beneficial treatment available for other forms of pulmonary hypertension such as PAH [2]. A further advantage of this study is the inclusion of 105 cases from different hospitals within the referral area, suggesting that these results are applicable across multiple centres.

Non-invasive identification of elevated PAWP using echocardiography and clinical assessment has been shown to be effective in discriminating Group 2 PH^27^. In Jacobs et al. [27], medical history, ECG and Echocardiography variables were combined to from a model to predict PH-LHD. This model was found to have an area under the curve of 0.93. As with this study Crawley et al. [28] demonstrated LA volume to be the most significant MRI derived variable to identify Group 2 PH though in a population of just those patients with IPAH and PH-HFpEF with AUC = 0.990, *p* < 0.001. A threshold of LA volume > 43 ml/m^2^ had 97% sensitivity and 100% specificity [28]. In contrast in our study of patients with suspected PH, MRI derived LA volume was of weaker accuracy (AUC = 0.844, *p* < 0.001) which was equivalent to CT derived LA area (AUC = 0.854, *p* < 0.001).

### 4.1 Limitations

A significant limitation is the retrospective study design of this study and potential selection bias. Prospective recruitment of patients to undergo, CT, MRI and RHC is advised to validate the findings of this study. In addition, evaluation in a second centre would be of value. This study was performed using non-cardiac gated CT pulmonary angiograms performed in the routine assessment of patients with suspected PH. ECG-gating may improve the repeatability and accuracy of LA area measurements [29]. The disadvantage of cardiac gated CT is it is associated with higher radiation dose [30] and is not routinely performed in patients with suspected pulmonary hypertension. Automated volumetric measurements of cardiac chambers have been used to predict Group 2 PH-LHD [31]. The use of automated volumetric chamber software is not in widespread use. Further studies evaluating such techniques in larger cohorts are warranted. Another limitation was 23% of the CTPAs were acquired from 66 different hospitals, so there was variation in the CT scanner used. This resulted in disparity between the slice thicknesses reconstructed at each centre and meant images were of varying quality, however this reflects a clinically relevant referral population. The number of patients with elevated PAWP is relatively small 88 with PAWP > 15 and 54 PAWP > 18, further study in larger patient cohorts likely in a multicenter setting is advised.

### 4.2 Clinical implications

There are broader clinical applications of the present study. CTPA is mainly done in emergency medicine. CT-derived LA area can easily be integrated in routine CTPA reporting. An estimate of PAWP, a surrogate of the left ventricular filling pressure, can guide towards more precise diagnosis. We do not propose LA area as a substitute for PAWP, rather a screening too to be used alongside a detailed clinical evaluation. As the main symptom of these patients is shortness of breath, appropriate further tests, including echocardiogram can help to confirm the diagnosis of LHD or heart failure with preserved ejection fraction.

## 5 Conclusions

CT derived left atrial area is a strong and independent predictor of increased PAWP in patients with suspected pulmonary hypertension. Enlarged left atrial area using thresholds of 26.8 cm^2^ and 30.0 cm^2^ to predict Group 2 pulmonary hypertension due to PAWP > 15 mm Hg and PAWP > 18 mm Hg respectively had a specificity of 89% and 94% in a validation cohort. In a subgroup analysis of only those patients with suspected PAH and PH-LHD, to identify PAWP>18 mm Hg a 30.3 cm^2^ threshold for LA area was 95% specific. The study demonstrates increased PAWP can be identified to a high degree of specificity using CT derived LA area.

## Supplementary data

Supplementary tables Image 1
