# Supplementary material for: CT derived left atrial size identifies left heart disease in suspected pulmonary hypertension: Derivation and validation of predictive thresholds
Source: Int J Cardiol. 2018 Jun 1;260:172–7. doi: 10.1016/j.ijcard.2018.02.114 (PMC5899969; doi:10.1016/j.ijcard.2018.02.114)
Supplement: Supplementary file 1 — Supplementary tables [file mmc1.docx]

**Supplementary Document**

**Supplementary Table 1:** Demographics Derivation vs Validation

|  |  | **Derivation Cohort**  **n=235** | **Validation Cohort**  **n=211** | **P Value** |
| --- | --- | --- | --- | --- |
| **Age** (years) |  | 64.1 (12.3) | 63.9 (13.5%) | 0.858 |
| **Sex** | **F** | 146 (62.1%) | 130 (61.6%) | 0.911 |
|  | **M** | 89 (37.9%) | 81 (38.4%) |  |
| **Body Surface Area** (m^2^) |  | 1.83 (0.24) | 1.83 (0.23) | 0.996 |
| **WHO functional Class** | **I** | 1 (0.4%) | 0 (0%) | 0.407 |
|  | **II** | 29 (12.6%) | 28 (13.5%) |  |
|  | **III** | 184 (80.0%) | 157 (75.8%) |  |
|  | **IV** | 16 (7.0%) | 22 (10.6%) |  |
| **ISWT Walking Distance** (m) |  | 231 (202) | 212 (186) | 0.332 |
| **Right Heart Catheter** | **mPAP** (mmHg) | 40 (14) | 41 (13) | 0.567 |
|  | **mRAP** (mmHg) | 10 (5) | 10 (6) | 0.137 |
|  | **PAWP** (mmHg) | 13 (5) | 13 (6) | 0.518 |
|  | **SVO_2_** (%) | 65.3 (8.9) | 65.1 (8.2) | 0.863 |
|  | **PVR** (dyn·s/ cm^5^) | 522 (390) | 538 (400) | 0.663 |
|  | **CO** (L/min) | 4.99 (1.59) | 4.86 (1.54) | 0.383 |
|  | **CI** (L/min/m^2^) | 2.73 (0.86) | 2.66 (0.79) | 0.868 |

Abbreviations: WHO-World Health Organisation, mPAP-mean Pulmonary Artery Pressure, mRAP-mean Right Atrial Pressure, PAWP-Pulmonary Arterial Wedge Pressure, SVO_2_-Mixed Venous Oxygen Saturation, PVR-Pulmonary Vascular Resistance, CO-Cardiac Output, CI-Cardiac Index, ISWT-Incremental Shuttle Walking Test,

**Supplementary Table 2-**Subgroup Analysis, Cohort demographic statistics.

|  |  | **Derivation Cohort**  N=119 | **Validation Cohort**  **n=112** | **P Value** |
| --- | --- | --- | --- | --- |
| **Age** (years) |  | 63.4 (13.3) | 62.9 (14.8) | 0.783 |
| **Sex** | **F** | 91 (76.6%) | 77 (68.8%) | 0.188 |
|  | **M** | 28 (23.5%) | 35 (31.3%) |  |
| **Body Surface Area** (m^2^) |  | 1.79 (0.23) | 1.82 (0.24) | 0.353 |
| **WHO functional Class** | **I** | 0 | 0 | 0.699 |
|  | **II** | 16 (13.9%) | 19 (17.3%) |  |
|  | **III** | 96 (83.5%) | 79 (71.8%) |  |
|  | **IV** | 3 (2.6%) | 12 (10.9%) |  |
| **ISWT Walking Distance** (m) |  | 244 (213) | 206 (193) | 0.205 |
| **Right Heart Catheter** | **mPAP** (mmHg) | 37 (15) | 39 (15) | 0.307 |
|  | **mRAP** (mmHg) | 10 (5) | 10 (6) | 0.335 |
|  | **PAWP** (mmHg) | 13 (5) | 14 (7) | 0.575 |
|  | **SVO_2_** (%) | 67.4 (9.61) | 65.7 (8.4) | 0.148 |
|  | **PVR** (dyn·s/ cm^5^) | 463 (425) | 493 (421) | 0.591 |
|  | **CO** (L/min) | 5.13 (1.73) | 4.97 (1.56) | 0.477 |
|  | **CI** (L/min/m^2^) | 2.88 (0.96) | 2.75 (0.77) | 0.251 |

Abbreviations: WHO-World Health Organisation, mPAP-mean Pulmonary Artery Pressure, mRAP-mean Right Atrial Pressure, PAWP-Pulmonary Arterial Wedge Pressure, SVO_2_-Mixed Venous Oxygen Saturation, PVR-Pulmonary Vascular Resistance, CO-Cardiac Output, CI-Cardiac Index, ISWT-Incremental Shuttle Walking Test,

**Supplementary Table 3:** Validation Cohort Sensitivity and Specificity of derived LA area limits at both PAWP>15mmHg and PAWP>18mmHg. (95% Confidence Intervals)

|  | **Threshold** (cm^2^) | **Sensitivity** | **Specificity** |
| --- | --- | --- | --- |
| **Full cohort (Validation)** |  |  |  |
| PAWP>15 | 26.8 | 60% (43-74%) | 89% (84-94%) |
| PAWP>18 | 30.0 | 53% (35-71%) | 94% (89-97%) |
| **Subgroup***  **(Validation)** |  |  |  |
| PAWP>15 | 27.1 | 65% (44-83%) | 90% (81-95%) |
| PAWP>18 | 30.3 | 60% (36-81%) | 95% (88-98%) |

Abbreviations: cm^2^-centimetres squared, PAWP-Pulmonary Arterial Wedge Pressure

***Patients excluded if moderate parenchymal lung disease, chronic or acute thromboembolic disease evident on CT angiography, or known diagnosis consistent with Group 5 pulmonary hypertension**

**Supplementary Table 4:** Intraclass Correlations results LA Area and LA Anterior-Posterior Diameter

| **CT Variable** | **Average Measures Inter-class Correlation Coefficient** | **P-Value** |
| --- | --- | --- |
| **LA Area** | 0.972 | <0.001 |
| **LA Anterior Posterior Diameter** | 0.921 | <0.001 |

Abbreviations: CT-Computed Tomography, LA-Left Atrium
